# Toward a fractalomic idiotype/anti-idiotypic paradigm

**DOI:** 10.6026/97320630018730

**Published:** 2022-09-30

**Authors:** Francesco Chiappelli, Jaden Penhaskashi

**Affiliations:** 1 Dental Group of Sherman Oaks, CA 91403 (www.oliviacajulisdds.com); 2 West Valley Dental Implant Center, Encino, CA 91316 (minimallyinvasiveperio.com)

**Keywords:** Corona Virus Disease 2019 (CoViD-19), Severe Acute Respiratory Syndrome Corona Virus-2 (SARS-CoV2), Artificial Intelligence (AI), Machine Learning (ML), Deep Learning (DL) Vaccinology, Fractal Analysis, Fractal Dimension (D), Idiotype, Immunoglobulin (Ig), T Cell Receptor (TcR), Complementarity-Determining Region (CDR)

## Abstract

The CoViD-19 pandemic has demonstrated the need for future developments in anti-viral immunology. We propose that artificial intelligence (AI) and machine learning, and in particular fractal analysis could play a crucial role in that context. Fractals - never-ending repeats of self-similar shapes whose composite tend to resemble the whole - are found in most natural biological structures including immunoglobulin and antigenic epitopes. Increased knowledge of the fractalomic properties of the idiotype/anti-idiotypic paradigm should help develop a novel and improved simplified artificial model of the immune system. Case in point, the regulation and dampening of antibodies as well as the synergetic recognition of an antigen by multiple idiotypes are both immune mechanisms that require further analysis. An enhanced understanding of these complexities could lead to better data analysis for novel vaccines to improve their sensitivity and specificity as well as open other new doors in the field of immunology.

## Background:

At the onset of the Severe Acute Respiratory Syndrome Corona Virus-2 (SARS-CoV2) pandemic, we proposed that artificial intelligence (AI) and computer-aided machine learning (ML) could be utilized to achieve new developments in anti-viral immunology in general, and vaccinology in particular. We suggested a bioinformation-driven approach in which through the integration of AI-aided tweening, a more complete understanding of all the events that collectively create the allostatic response to a viral infection, as well as the cumulative negative factors that resist said allostasis could be developed. This enhanced understanding of immunity was posited to in-turn lead to a more effective incorporation of useful data into new artificial immunization techniques. Today, the Corona Virus Disease 2019 (CoViD-19) has demonstrated resistance against global efforts to slow its spread. It is more critical than ever to conceive, develop and evaluate antigen tests with optimal sensitivity and specificity that can be implemented for quick and reliable diagnosis, and vaccines, although reducing hospitalization, prove to not be as effective against emerging strains of the virus. The need for further innovations and discoveries in immunology is timely and critical. There is unquestionable international urgency for the development, evaluation and wide distribution of low-cost, high accuracy diagnostic testing tools and vaccination protocols with fully characterized cross-reactivity with any variants of future viruses, within the upcoming decades [1-4].

## Description:

Studies have recently emerged that supplement our original model within the relevancy of CoViD-19 - artificial intelligence correcting gaps of knowledge regarding the correlation between relevant datasets and individual/collective resistance against the virus. Case in point, machine learning (ML), designed to learn automatically from patterns in databases, has been found to effectively assist with CoViD-19 diagnostics when fed biological markers. By contrast, the reports have shown the more novel deep learning model, designed to resemble the human thinking process with even greater potential effectiveness of providing relevant information on CoViD-19 cases [5,6]. Specific algorithms can be generated by both ML, which is a type of computer-mediated AI that allows software applications to become more accurate at predicting outcomes without being explicitly programmed to do so, and deep learning (DL), a specific sub-type of ML constricted and designed to imitate (or to approximate) the way humans gain certain types of knowledge, including that derived from statistics, statistical inference and predictive modeling. Both ML and DL algorithms are increasingly utilized for the improvement of national public health monitoring protocols and with real-time forecasting of local epidemics in a variety of countries. Some recently tested DL models indeed suggest potential effective anti-virals and vaccine candidates for combating SARS-CoV-2 [5-7], and may be presently utilized against the emerging monkeypox epidemics.

It is possible and even likely that fractal analysis is one distinct aspect of AI with great potential in the future of immunology and more specifically of vaccinology. This is conceptualized by Benoit B. Mandelbrot as *"... rough or fragmented geometric shape"* which when split into multiple smaller components, each individually resemble the shape of its self (i.e., self-similarity) and of the whole. Geometric fractals are said to have been recognized since the start of the seventeenth century with German scientist, Gottfried Wilhelm von Leibniz's discourse on the idea of recursive self-similarity and constant use of the term *"fractional exponents"*. The fractal dimension D, a ratio that is essentially a measure of the amount of space an object's fractal pattern will fill, typically derived by the Box counting approach, is used as an index of complexity of the object being observed [8-13]. Fractal dimensions are seen in most complex natural phenomenological structures and have been recognized to have potential practical applications in numerous areas of biology and medicine, including immunology. By scaling consistent self-similarity characteristics and discordant irregularity parameters through fractal analysis, utilizable information about the fractalomic properties of the object of study emerges [14-33]. Case in point, we have previously established that yielding further fractalomic information from the molecular moieties and signatures of structural proteins in premalignant and oral squamous cell carcinoma could help lead to the future detection of said molecular signatures and in turn, enable a quicker diagnosis of said carcinoma. Alternatively, in regards to the advancement of immunology, analyzing the fractalomic properties of a given antibody *répertoire* could assist with enhancing a generated vaccine's stability, sensitivity and response time [15, 16, 20, 34].

The question then becomes, in what way can fractal analysis of the immune system be integrated with AI and ML to generate a more comprehensive understanding of the specific immune response, and more specifically of vaccinology? The required connecting factor lies within the postulated idiotype/anti-idiotypic paradigm, briefly outlined below. In brief, the idiotype is the variable sequence and region (V) on the immunoglobulin (Ig) or T cell receptor (TcR). It reflects a molecule structure specific to its antigen specificity. The complete collection of Ig's and TcR's that target a certain antigen is labeled as the idiotype family of an organism. It is the entire idiotype working together that enables polyvalent antigen specificity against the pathogen. Sequences of amino acids within the V region of an immune molecule, complementarity-determining regions (CDRs), determine its antigen specificity, and therefore its idiotype [35]. According to Geoffrey W. Hoffman's symmetrical immune theory [38], an elaboration on Niels Jerne's model [36,37], a typical immune response comprises of both antigen-specific and anti-idiotypic antibodies. The latter are designed to regulate the response through suppressing and eventually terminating the antibody-mediated immune response. In this idiotype/anti-idiotype paradigm, Igs will target the specific antigen, but also act as an antigen itself, leading the production of anti-antibodies. The resulting well-regulated response to the antigen controls and modulates switching between different states of immuno biological homeostasis, variably suppressed immune activation, as well as an allostasis that is directed back towards homeostatic balance [36-41,43].

Each Ig, and therefore, each collective idiotype and anti-idiotype of the idiotype/anti-idiotypic network, has distinct fractalomic properties that can be quantified through proper fractal analysis. To recognize and respond to a non-self or self-antigen, immune cells work in concert - the collective organism’s idiotype binding to the antigen due to sharing certain surface receptor molecules; it is also theorized that this multi-site recognition is more reliable for distinguishing between antigens than single site recognition. Once the fractalomic properties of the symmetrical idiotype/anti-idiotype paradigm is quantified, a simplified artificial model of immune multi-site recognition could then potentially be developed by ML, in turn yielding increased comprehension of auto-immune disorders as well as enhanced data analysis during vaccine development and testing [42-47]. New and improved artificial immune network models currently under development and testing operate through the induction of the binding of an antigen to its idiotype, which then yields a network structure that is further analyzed. This approach enhances the recognition and analysis of new binding clusters in the data stream, and serves in the validation of critical quantitative fractalomic descriptors of antigen/antibody binding complexes, including their fractal dimension, D [35,45-47].

## Conclusion:

As AI in general and ML and DL in particular continue to expand and develop, our ability to analyze fractolomic properties and characteristics of antigen-idiotype interactions will improve. The continued integration of ML and DL with modern fractal analysis will ensure complexities of the immune response. Together, these novel viewpoints will increase the efficiency predicted increase in the specificity, strength, and overall efficiency of novel developed vaccines with correlated enhancement of vaccinology data analysis techniques [3, 49]. Recent discoveries in immunology have shown a similar progression to the one postulated, specifically for understanding the antibody response to antigens of parasite origin. Through the identification of previously unknown critical targets of antigen-idiotype interactions and of a complex diversification mechanism, new possibilities for passive antibody therapies and vaccine design have begun to emerge that provide further sterilizing immunity [50]. Taken together, the lines of evidence discussed here demonstrate that applying the fractalome to understand the complex immune response better and more in-depth will likely lead to new and improved developments in vaccinology. As noted above, Perelson and Oster (1979) [44] discussed how the probability of recognizing a novel antigen will increase as its antibody *répertoire* increases, as idiotypes can work together to respond to an abundance of antigens, depending on the extent that a particular region on the novel antigen aligns with the CDR of a certain idiotype. We have proposed here that through fractal analysis, the dynamic relationship between idiotypes and CDRs can be further understood [44, 45] - potentially allowing for the creation of more versatile vaccines that can lead to an effective immunity against multiple strains of a virus - a possible solution to the constant evolution of the CoViD-19 virus in recent years.
